# The proactive bilingual brain: Using interlocutor identity to generate predictions for language processing

**DOI:** 10.1038/srep26171

**Published:** 2016-05-13

**Authors:** Clara D. Martin, Monika Molnar, Manuel Carreiras

**Affiliations:** 1 BCBL. Basque Center on Cognition, Brain and Language, Paseo Mikeletegi 69, 20009 San Sebastian, Spain; 2 IKERBASQUE, Basque Foundation for Science, Maria Diaz de Haro 3, 48013 Bilbao, Spain

## Abstract

The present study investigated the proactive nature of the human brain in language perception. Specifically, we examined whether early proficient bilinguals can use interlocutor identity as a cue for language prediction, using an event-related potentials (ERP) paradigm. Participants were first familiarized, through video segments, with six novel interlocutors who were either monolingual or bilingual. Then, the participants completed an audio-visual lexical decision task in which all the interlocutors uttered words and pseudo-words. Critically, the speech onset started about 350 ms after the beginning of the video. ERP waves between the onset of the visual presentation of the interlocutors and the onset of their speech significantly differed for trials where the language was not predictable (bilingual interlocutors) and trials where the language was predictable (monolingual interlocutors), revealing that visual interlocutor identity can in fact function as a cue for language prediction, even before the onset of the auditory-linguistic signal.

The human brain is continuously generating predictions in specific and controlled situations, such as visual perception^1^,^2^. However, it is still unclear whether it also relies on predictions in ‘high level’ tasks, such as language perception. Here we ask whether bilingual speakers capitalize on natural cues (such as interlocutors) to predict (or pre-activate) a target language efficiently. Current models of spoken language perception, including models developed for both monolingual and bilingual listeners, do not include prediction of linguistic information based on non-linguistic cues *before* speech onset. Rather, the models of bilingual spoken language perception generally promote a reactive control behind an initial state of non-selectivity. Specifically, the two languages of the bilingual listeners are initially co-activated until clear linguistic information (e.g., a phoneme) indicates the appropriate language^3^,^4^,^5^. Thus, the models mainly focus on reactive control without considering the role of proactive control (prediction) before speech onset. In contrast, here we show that language activation in perception can occur even before the onset of speech, because listeners can use visual interlocutor identity as a cue to *predict* a language prior to the occurrence of any explicit linguistic event.

Using event-related potentials (ERPs), we measured bilinguals’ brain responses before interlocutors produced speech. The ERP technique offers an exceptional window to investigate the time course of cognitive events, allowing us to capture brain responses before and after speech onset. In particular, we capitalized on this technique to investigate, for the first time, the influence of interlocutors on brain activity before any acoustic signal. Participants were first familiarized with monolingual and bilingual interlocutors (six female interlocutors presenting themselves to the participant, two of them speaking only in Basque, two of them speaking only in Spanish and two of them using alternatively the two languages (the role, monolingual or bilingual, and the order of presentation were counterbalanced). Then, participants completed an audio-visual lexical decision task (test phase) in which the interlocutors uttered lexical items (70% of words/30% of pseudowords). Critically, the speech onset started about 350 ms after the beginning of the video. Therefore, if visual interlocutor identity functions as a cue for prediction of the language, changes in the ERPs are expected in the absence of any acoustic/linguistic signal, that is, even before an interlocutor starts to speak. In particular, brain responses time-locked to video onset (pre-speech activity) should differ between monolingual and bilingual interlocutors (predictable vs. unpredictable conditions), meaning that participants are sensitized to the predictability of linguistic information when facing a monolingual interlocutor but not when facing a bilingual interlocutor.

Further, this paradigm also allowed us to investigate the effect of such language prediction on speech processing itself (post-speech activity). If language preparation is different in a monolingual and bilingual interlocutor context, this might potentially have consequences on speech perception and thus influence ERPs after speech onset. In the test phase, participants performed a lexical decision task on words and pseudo-words produced by the monolingual and bilingual interlocutors. The influence of interlocutor identity during speech processing was assessed by exploring the N1 and N400 components (time-locked to audio onset). The N400 ERP response is typically modulated by the lexical status of stimuli in auditory lexical decision tasks^6^,^7^,^8^. This component is usually larger in amplitude when processing a non-word or pseudo-word relative to a real word^8^. The N400 effect (difference in mean amplitude between pseudowords and words) reflects lexical integration efficiency. The more efficient word/pseudoword discrimination is, the larger the N400 effect is, and the faster the reaction times are in a lexical decision task^9^. If participants can predict a given language when facing a monolingual – but not a bilingual interlocutor –, we expect lexical integration to be easier. Thus, the N400 effect should be observed in both interlocutor contexts (reflecting lexical integration), but a larger N400 effect is expected when participants process items uttered by monolingual (reflecting more efficient word/pseudoword discrimination). The N1 ERP response codes stimulus-specific features likely to be critical for speech perception^10^, and lexicality effects were also previously reported in such early time-windows when lexical access is facilitated and takes place very early in time after stimulus display^11^,^12^. If participants can predict a given language when facing a monolingual – but not a bilingual interlocutor –, we hypothesize that lexical access should be facilitated and speeded up. Thus, the N1 early component should be modulated by lexicality when facing a monolingual and not a bilingual interlocutor. Those N1 and N400 patterns would suggest that lexicality effects arise earlier (in earlier ERP components) and are stronger (larger N400 effects) when facing a monolingual compared to a bilingual interlocutor. We also hypothesize that reaction times in the lexical decision task (deciding whether an item is a real word or a pseudo-word) should be faster in monolingual than in bilingual interlocutor contexts.

In sum, we tested whether listeners who are proficient users of two languages can use interlocutor identity as a non-linguistic cue for language prediction before the onset of any speech signal, and whether this prediction influences later lexical access and integration, as measured with a lexical decision task during speech processing.

## Results

### ERP results

**Figure 1:**
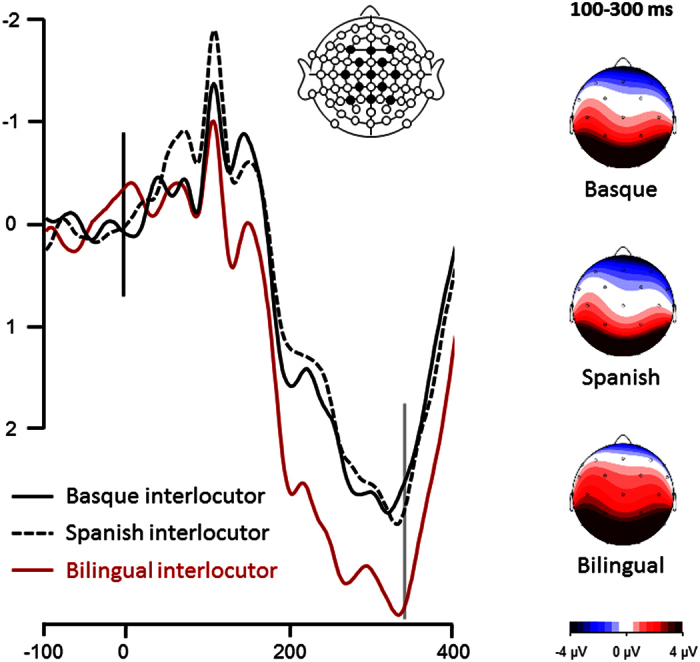
Event-related potential results for the interlocutor identity effect (pre-speech analysis). Time zero (black vertical line) indicates the presentation of the video of the interlocutor. Time 350 (grey vertical line) indicates the averaged onset of speech. Black line depicts ERPs measured for Basque identity (interlocutors speaking only in Basque during familiarization); Dotted black line depicts ERPs measured for Spanish identity (interlocutors speaking only in Spanish during familiarization); Red line depicts ERPs measured for bilingual identity (interlocutors speaking in both Basque and Spanish during familiarization). Linear derivation of F3, F4, FC1, FC2, Fz, C3, C4, Cz, CP1, CP2, Pz, P3, P4 is presented. Negativity is plotted up. Scalp distributions of the ERPs in the [100–300] ms time-window are shown on the right side of the figure, for each of the three conditions.

**Figure 2:**
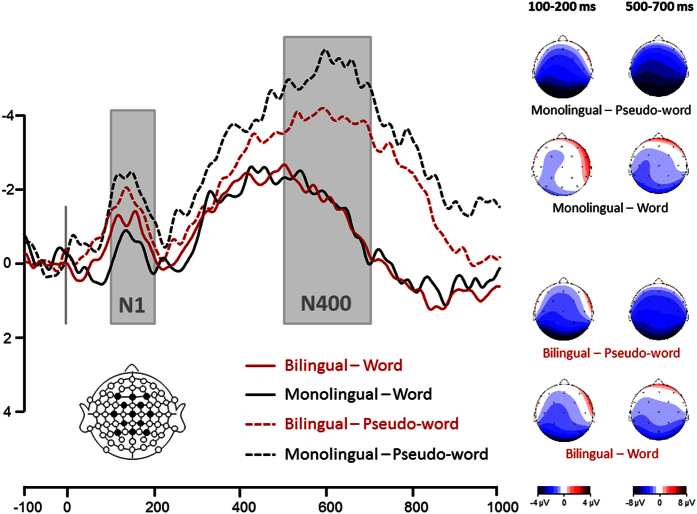
Event-related potential results for the lexicality x interlocutor context interaction (post-speech analysis). Time zero (grey vertical line) indicates the speech onset. Red line depicts ERPs measured for words uttered by bilingual interlocutors. Black line depicts ERPs measured for words uttered by monolingual interlocutors. Dotted red line depicts ERPs measured for pseudo-words uttered by bilingual interlocutors. Dotted black line depicts ERPs measured for pseudo-words uttered by monolingual interlocutors. Linear derivation of F3, F4, FC1, FC2, Fz, C3, C4, Cz, CP1, CP2, Pz, P3, P4 is presented. Negativity is plotted up. Scalp distributions of the ERPs in the [100–200] and [500–700] ms time-windows are shown on the right side of the figure, for each of the four conditions.

Two main analyses were conducted on ERP results: (1) an ANOVA to investigate the ‘pure’ effect of interlocutor identity (with no influence of speech) by comparing Basque vs. Spanish vs. bilingual interlocutor identities, from the onset of the video up to the averaged onset of speech (in three windows [0–100], [100–200] and [200–300] ms on ERPs time-locked to video onset; see Table 1 and Figs 1 and 2) a 2 × 2 ANOVA with interlocutor status (monolingual vs. bilingual) and lexicality (words vs. pseudowords) as within-subject factors, to investigate the effect of interlocutor status after speech onset, during lexical access/integration (ERPs time-locked to audio onset; see Table 2 and Fig. 2).

The main outcome of the first analysis was that the influence of interlocutor identity on the participant’s brain responses started around 100 ms after the onset of the video, even before the onset of speech (see Fig. 1). Between 100 and 300 ms after the onset of the video, participants processed the videos differently for bilingual and monolingual interlocutors.

The second important result was that the lexicality effect was significant in the N1 time-window in the monolingual interlocutor context but not in the bilingual interlocutor context (see Fig. 2). Later on, words and pseudo-words were processed differently in the N400 time-window in both contexts, and the lexicality effect was larger in monolingual than bilingual interlocutor context.

We also computed correlations between ERP mean amplitudes pre- and post-speech. In both monolingual and bilingual interlocutor contexts, ERP mean amplitudes in the [100–200] ms pre-speech time-window were significantly correlated with the magnitude of the lexicality effect (ERP mean amplitude for words minus pseudo-words) in the post-speech N400 time-window (Pearson correlations: monolingual context: −0.50; p = 0.017; bilingual context: −0.44, p = 0.04).

### Behavioral results

In order to investigate the potential behavioral consequences of language prediction, two analyses were conducted on reaction times (measured from the onset of the video segment; see Table 3).

A 2 × 2 ANOVA investigating the interlocutor (monolingual vs. bilingual context) and lexicality (words vs. pseudo-words) effects revealed a main effect of interlocutor context (F[1,22] = 8.67, p = 0.008), showing that participants were slower in responding to trials uttered by bilingual interlocutors compared to monolingual interlocutors. There was also a main effect of lexicality (F[1,22] = 89.21, p < 0.001), showing that participants were faster to respond to words than pseudowords in the lexical decision task^13^. The two factors did not interact (F[1,22] = 2.76, p = 0.11).

We also computed correlations between pre-speech ERP mean amplitudes and reaction times. In both monolingual and bilingual interlocutor contexts, ERP mean amplitudes in the [100–200] ms pre-speech time-window were correlated with the magnitude of the behavioral lexicality effect (reaction times for words minus pseudo-words; Pearson correlations: monolingual context: 0.52; p = 0.013; bilingual context: 0.42, p = 0.05).

## Discussion

Our main results suggest that listeners can rely on interlocutors as a cue for predicting linguistic information. Specifically, as illustrated in Fig. 1, proficient bilingual listeners exhibit different neural patterns when they see an interlocutor whose language can be predicted (monolingual interlocutor), as opposed to seeing an interlocutor whose language cannot be predicted (bilingual interlocutor). This finding has important consequences for models of spoken language perception, because it demonstrates that (1) non-linguistic contextual information is integrated into linguistic processing mechanisms by means of prediction; (2) language activation in bilingual listeners can occur prior to any linguistic input, even though most current models of bilingual perception ignore such a possibility^3^,^4^,^5^.

Our results thus suggest that listeners can exploit interlocutor identity during language processing to predict linguistic information. A growing body of research suggests that the brain takes advantage of frequent and known situations in the environment to anticipate upcoming events^1^,^2^. Thus, the proactive human brain could be predictive in a wide variety of domains, including language processing^1^. In fact, prediction has already been observed during sentence comprehension^14^,^15^. Importantly though, the present study demonstrated that prediction during language processing can occur even before any linguistic input is perceived.

Predicting the language that will be used next should certainly improve further speech perception^16^,^17^. Analyses of ERPs and reaction times post-speech corroborate this assumption. When listeners were able to prepare for a language before speech onset (when facing a monolingual interlocutor), lexical access and integration were facilitated as compared to when they could not get prepared (when facing a bilingual interlocutor). The ERP lexicality effect emerged earlier (N1 time-window) and was larger (N400 time-window) in the monolingual compared to the bilingual context. In fact, the N1 component was significantly larger for pseudo-words than for words^11^,^12^,^18^,^19^,^20^ only in the monolingual interlocutor context. Later, in the [500–700] ms time-window, pseudo-words elicited a larger N400 component than words^6^,^7^,^8^,^9^ in both interlocutor contexts. Critically, this N400 lexicality effect was larger in the monolingual compared to the bilingual interlocutor context (reflecting more efficient word/pseudoword discrimination). Furthermore, reaction times were faster in the monolingual compared to the bilingual interlocutor context.

This facilitation in lexical access/integration during speech processing supports recent findings showing that attentional resources or intentionality may trigger proactive top-down signals which enhance lexical processing both in language comprehension^21^,^22^ and production^23^,^24^ (see^25^ for bilingual production). It also supports previous studies showing significant effects of contextual information at the lexical level, by using linguistically or culturally biased interlocutor faces^17^,^26^,^27^. The important advance here is that, based on pre-speech analyses, we can argue that this facilitation in lexical access and integration stems from language prediction performed before speech onset. This is corroborated by the fact that lexicality effects (both in ERPs and reaction times) were significantly correlated with mean amplitudes in the pre-speech [100–200] ms time-window. Altogether, these data strongly suggest that language prediction based on interlocutor identity took place before speech onset, and that it facilitated further lexical processing during speech perception.

This observation of language prediction based on interlocutor identity has relevant implications for models of bilingual language control. The best fits with our findings appear to be the Bilingual Interactive Activation Model of Lexical Access (BIMOLA^28^), specifically developed for bilingual speech perception, and the Bilingual Interactive Activation + model (BIA+^29^). Those models allow top-down pre-activation (or modulation) of languages based on contextual information. In the BIMOLA, interlocutor identity (contextual information) should bias language selection in a top-down manner at the lexical level, as indicated by the present results^17^,^26^,^27^. The BIA+ model includes a ‘task schema’ level which processes information from the extra-linguistic context and modulates language processing and/or decision making, based on the subjects’ expectations, which is also supported by the present results. Importantly, the novelty of our study is to show that models of bilingual word recognition should consider that selective language preparation can take place even *before* any linguistic input, based on ‘naturalistic’ cues such as an interlocutor identity. Most accounts neglect the role of contextual top-down influences before any linguistic input but our study shows that not only reactive but also proactive control mechanisms may play an important role in language control.

It could be also argued that the interlocutor identity effects observed in the pre-speech time-window are the consequence of task difficulty/complexity. In fact, the pre-speech ERP pattern observed in the present study (see Fig. 1) can be interpreted in terms of P3b modulation: proactive control in the interval between cues and targets has been shown to elicit a positive component (P3b), reaching its maximum around 300 ms after cue onset^30^, and reflecting context updating and task goal activation^31^. We argue that higher P3b amplitude for bilingual compared to monolingual interlocutor cue here characterizes greater engagement of proactive control, since bilingual cues need more complex context updating and task goal pre-activation (no possibility of preparing one of the two languages). Nevertheless, the P3b component is known to be larger for more salient cues. Therefore, the present pre-speech P3b component might be caused by the less frequent (more salient) bilingual interlocutor context as compared to monolingual interlocutor context and thus by preparation for a more difficult task. However, the correlations of the pre-speech ERP patterns with post-speech lexicality effects considerably strengthen the language prediction interpretation of the pre-speech interlocutor effect. In any case, even if the pre-speech ERP modulations only reflect preparation to tasks of different difficulty, the important predictive result still holds, given that task preparation has an impact on the speech processing that follows (lexicality effect in N1, larger N400 lexicality effect and faster reaction times).

Therefore, the present data align better with the main conclusion that interlocutor identity is a cue used by bilingual listeners for task preparation, and that this preparation for a monolingual or bilingual language context influences further speech processing. We suggest that task preparation takes place during the display of the interlocutor’s face (cue). A more complex context updating and task goal preparation is reflected by a larger pre-speech P3 amplitude^32^. We also argue that a more difficult task preparation during the pre-speech phase is associated with a more difficult lexical access and integration during the post-speech phase. This means that a larger pre-speech P3 amplitude should be associated to a smaller post-speech N400 lexicality effect (harder word/pseudoword discrimination) and to longer RTs in lexical decision, indicating that the P3 amplitude is predictive for subsequent task performance^32^. In the present study, we show that task preparation is complex when facing a bilingual interlocutor (no possible anticipation of the language to come), which makes post-speech language processing harder. This is reflected by a larger pre-speech P3 component, which correlates with a smaller post-speech N400 lexicality effect (poor word/pseudoword discrimination) and with larger RTs in the lexical decision task. This is, to our knowledge, the first study showing how non-linguistic cues are used in ‘naturalistic’ conditions for language preparation. This result is even more striking after considering that the participants were familiarized with the interlocutors for only a few minutes, suggesting fast development of language-interlocutor associations.

In sum, the current experiment demonstrated, for the first time, that prediction of linguistic information in bilinguals can occur without any explicit linguistic input, based on non-linguistic cues alone. These finding support the possibility that the linguistic system, in the case of monolingual interlocution, can also exploit non-linguistic interactional information during linguistic processing. In fact, adding proactive control to language control models should not be restricted to bilingual language processing and has to be extended to monolingual speech perception theories (see^33^ for an attempt at integrating active control systems in speech perception models).

## Material and Methods

### Participants

Thirty early proficient Spanish-Basque bilinguals took part in the experiment. Seven participants were removed from the group because of a large number of rejected epochs in the ERP data analysis (more than 50% of rejected epochs). All participants (N = 23; mean age = 24.2 ± 5.4; 14 female) started to acquire Spanish and Basque before age 3 (median AoA for Spanish 0 years old, range 0–2; for Basque 0 years old, range 0–3), and rated their overall Spanish and Basque proficiencies to be native-like and comparable (mean scores of 9.7 ± 0.5 and 9.4 ± 0.5 for Spanish and Basque respectively on a 0–10 scale). Objective scores of participants’ language proficiency were measured through a 77 picture-naming task (average Spanish score: 76.5 ± 0.8; average Basque score: 75.3 ± 1.8). Also, participants reported using both of their languages on a daily basis with family, friends, and colleagues. The experiment was performed in accordance with relevant guidelines and regulations and was approved by the BCBL ethic committee. Informed consent was obtained from all subjects.

### Material and procedure

#### Familiarization phase

First, participants were presented with 12 short video segments (approx. 2 minutes each; 2 video segments per interlocutor) of six female interlocutors presenting themselves to the participant (speaking about family, hobbies, work etc.). Two interlocutors were speaking only in Basque (Basque monolingual interlocutors), two were speaking only in Spanish (Spanish monolingual interlocutors) and two presented themselves by using alternatively the two languages (Spanish-Basque bilingual interlocutors). The bilingual interlocutors code-switched (when it was natural to do so within a sentence), but also occasionally used full Spanish or Basque sentences within one video segment. The role (monolingual or bilingual) and the order of presentation of each interlocutor were counterbalanced across participants. This way, we made sure that any interlocutor effect could only be due to her language status (monolingual or bilingual). During this familiarization phase, participants were implicitly learning the association between each interlocutor and their language (Spanish, Basque or bilingual). To ensure that the participants paid attention to the video clips, their comprehension was assessed by true/false questions after each video segment.

#### Test phase (Lexical decision task)

After the familiarization, an audio-visual lexical decision task was presented to the participants. They were instructed that they were going to listen to Basque and Spanish words uttered by the 6 interlocutors they were just familiarized with. They had to decide if the word they heard was real or not in any of the languages (Basque or Spanish) by pressing a corresponding button (counterbalanced across participants). Each participant was presented with 150 Spanish words, 150 Basque words, 60 Spanish-like pseudowords and 60 Basque-like pseudowords. Spanish and Basque non-cognate words were selected from the Spanish B-Pal^34^ and Basque E-Hitz^35^ word databases. Spanish and Basque words were all nouns, matched on syllable length (2 to 4 syllables; Spanish words: 2.99; Basque words: 2.99; p = 1), number of neighbors (Spanish words: 1.5; Basque words: 1.4; p = 0.87), age of acquisition (Spanish words: 3.2; Basque words: 3.3; p = 0.61), concreteness (Spanish words: 4.06; Basque words: 4.11; p = 0.85) and frequency per million (Spanish words: 73; Basque words: 71; p = 0.89). Homographs and homophones were excluded. The average duration of the words was 715 ± 140 ms. The 120 pseudo-words were constructed by replacing a maximum of two phonemes from real Spanish and Basque words. The average duration of the pseudowords was 734 ± 155 ms. Video segments of the interlocutors pronouncing the Spanish and Basque words and pseudowords were created for the test phase. Videos were edited in such way that there was an average of 345 ± 87 ms gap between the onset of the video and the onset of the auditory signal. There were no significant differences between the gap durations of Basque vs. Spanish words or words vs. pseudowords (all ps >0.05). Note that another manipulation was initially added in the design: The monolingual interlocutors produced congruent and incongruent words (in a different language that was used in the familiarization phase). Out of the scope of the present focus on language prediction, this manipulation is not presented in the present article. Still, the congruent vs. incongruent status of the videos were counterbalanced across participants.

During the test phase, participants were first presented with a fixation cross at the center of the screen for 800 ms. The cross was followed by a video segment showing an interlocutor producing a word or a pseudoword. The participant then had to press one keyboard button or another, according to whether the stimulus was a real word (either Spanish or Basque) or a pseudoword. The answer triggered the next trial. Each monolingual interlocutor produced randomly 75% of the real words congruently (e.g., Spanish word for Spanish monolingual interlocutor) and 25% of them incongruently (e.g., Basque word for Spanish monolingual interlocutor). Note that the incongruent trials were added for two main reasons (1) to mimic the situation of bilingual environments, such as the Basque Country, in which interlocutors are hardly ever monolinguals. Monolinguals speak mainly in their first language, but occasionally use one word in the other language, since they have some knowledge of the other language. (2) The second reason was to have a post-speech control that the participants were sensitive to the interlocutors in case the effect was not captured by the differences in the N400 (post-speech) or even in the pre-speech. Importantly, we captured effects both post- and pre-speech. Nevertheless, to verify whether the effect of the incongruent trials changed as the experiment developed, we analyzed separately the first and the second half of the experiment. We computed the ERP patterns on the first 210 trials preceding the break in the middle of the experiment, and the ERP patterns on the last 210 trials (second half of the experiment after the break). We observed that the effects were not modulated by the incongruent trials. Visual inspection and statistical analyses revealed that the pre-speech ERP pattern was similar in the first and second half of the experiment (similar ERP waves for Basque and Spanish interlocutor conditions and larger positivity for bilingual interlocutor condition). Thus, the monolingual or bilingual status of the interlocutor was preserved despite the occurrence of the incongruent trials.

Congruent and incongruent trials were distributed in a 75–25 percentage ratio to maintain the monolingual status of interlocutors during the task. For the same reason, all pseudowords were congruent. Congruent trials were randomized in such a way that the same interlocutor could appear a maximum of three times in a row. Incongruent trials were randomized so that two or more incongruent trials could not appear in a row. Bilingual interlocutors produced Spanish and Basque words 50–50% of the time. Since bilingual interlocutors produced only congruent trials (both languages used during the familiarization phase), congruent trials only were used in the analyses presented here. We considered Spanish and Basque words and pseudowords uttered by bilingual interlocutors, Spanish words and pseudowords uttered by Spanish monolingual interlocutors and Basque words and pseudowords uttered by Basque monolingual interlocutors. This way, words and pseudo-words uttered by monolingual and bilingual interlocutors could be directly compared in a 2 (lexicality) ×2 (interlocutor context) design. The two Spanish interlocutors uttered 75 Spanish words and 40 Spanish-like pseudoword. The two Basque interlocutors uttered 75 Basque words and 40 Basque-like pseudowords. The two bilingual interlocutors uttered 50 Spanish words, 50 Basque words, 20 Spanish-like pseudowords and 20 Basque-like pseudowords. The congruency effect (i.e., difference in processing congruent and incongruent words; see^17^) is not reported here since it is not relevant for the purpose of the current paper.

### EEG recording and processing

The EEG signal was recorded in reference to the left mastoid at a rate of 500 Hz from 27 electrodes mounted in an elastic cap according to the 10–20 convention. Vertical and horizontal EOG were recorded simultaneously with the EEG. Impedances were kept below 5 kOhm. EEG activity was filtered off-line: 0.1 Hz (12 dB/oct) low cutoff and 30 Hz (48 dB/oct) high cutoff. Eye blink artifacts were corrected using Gratton & Coles’ procedure^36^, implemented in Brain Vision Analyzer 2.0 (Brain Products, München, Germany), and any remaining artifacts were manually dismissed. For pre-speech analyses, epochs ranged from −100 to 300 ms, time 0 ms being the onset of the video segment. For post-speech analyses, epochs ranged from −100 to 1000 ms, time 0 being the onset of the audio (around 350 ms after video onset). Baseline corrections were performed in reference to pre-stimulus activity (from −100 to 0 ms; 0 ms being the video onset in pre-speech analyses and the audio onset in post-speech analyses) and individual averages were digitally re-referenced offline to the mean of left and right mastoid signals. Segments were averaged for each participant, each experimental condition and each electrode. Since we did not have a specific hypothesis about the topography and latency of the interlocutor identity effect in the pre-speech analysis, mean amplitudes were measured in the time-window following the video onset (0 to 300 ms), in sequential 100-ms non-overlapping time-windows from the onset of the video, resulting in three time-windows. Regarding post-speech analyses, mean amplitudes were measured in the N1 (100–200 ms) and N400 (500–700 ms) time-windows following the audio onset. Since we did not have strong hypotheses regarding the topography of the ERP patterns, especially for pre-speech analysis, the signal was analyzed over the 13 more central electrodes, including Cz and neighboring electrodes: F3, Fz, F4, FC1, FC2, C3, C4, CP1, CP2, P3, Pz, P4. This way, ERP modulations were captured over the entire scalp, with no a priori on localization, excluding the most external electrodes usually more noisy.

## Additional Information

**How to cite this article**: Martin, C. D. *et al.* The proactive bilingual brain: Using interlocutor identity to generate predictions for language processing. *Sci. Rep.* **6**, 26171; doi: 10.1038/srep26171 (2016).

## Figures and Tables

**Table 1:** Results of the ANOVA exploring interlocutor identity effects in three 100 ms time-windows (pre-speech analysis).

	Main effect		Post-hoc analysis		
	Identity		‘Bil – Ba’	‘Bil – Sp’	‘Ba – Sp’
	F	*p*	*p*	*p*	*p*
0–100 ms	3.09	0.07			
100–200	**6.11**	**0.008**	**0.003**	**0.03**	0.59
200–300	**4.09**	**0.03**	**0.05**	**0.02**	0.70

Main identity effect is presented on the left panel (Spanish vs. Basque vs. bilingual interlocutors). The three possible post-hoc contrasts (Bonferroni tests) are presented on the right panel. All analyses reported have [1,21] as degrees of freedom and N = 23. Bil = Bilingual speaker; Ba = Basque speaker; Sp = Spanish speaker. Significant values are in bold.

**Table 2:** Left panel: Results of the ANOVA comparing interlocutor (monolingual vs. bilingual context) and lexicality (words vs. pseudo-words) effects in the N1 time-window (100–200 ms).

		N1		N400	
		F	*p*	F	*p*
ANOVA	Interlocutor effect	0.38	0.54	0.30	0.59
	Lexicality effect	**16.55**	**<0.001**	**68.24**	**<0.001**
	Interaction	**5.42**	**0.03**	**5.42**	**0.03**
Post-hoc analysis	Lexicality effect in monolingual context		**0.001**		**<0.001**
	Lexicality effect in bilingual context		1.00		**0.004**

**Right panel:** Results of the ANOVA comparing interlocutor and lexicality effects in the N400 time-window (500–700 ms). All analyses reported have [1,22] as degrees of freedom and N = 23. Post-hoc analyses = Bonferroni tests. Significant values are in bold.

**Table 3:** Behavioral results for the interlocutor and lexicality effects.

	Reaction times	
	Mean	*SD*
Bil – PW	1767	328
Bil – W	1567	335
Mono – PW	1738	325
Mono – W	1544	341

Bil = bilingual interlocutor; Mono = monolingual interlocutor; PW = pseudo-word trial; W = word trial.
